# The immune microenvironment of the central nervous system and neuromodulation: new perspectives in lung cancer brain metastases research

**DOI:** 10.3389/fimmu.2026.1763830

**Published:** 2026-04-29

**Authors:** Xiaoyu Yang, Chenxing Huo, Jianhui Tian, Zhiqi Xiong, Jialiang Yao, Bin Luo

**Affiliations:** 1 Department of Oncology, Shanghai Municipal Hospital of Traditional Chinese Medicine, Shanghai, China; 2 Institute of Oncology, Shanghai Municipal Hospital of Traditional Chinese Medicine, Shanghai, China; 3 Center for Excellence in Brain Science and Intelligence Technology, Institute of Neuroscience and State Key Laboratory of Neuroscience, Chinese Academy of Sciences, Shanghai, China

**Keywords:** brain metastases, central nervous system, immunity, lung cancer, tumor microenvironment

## Abstract

Brain metastases (BM) from lung cancer remain a devastating complication that severely compromises patient survival. Its development is driven by dynamic and complex interactions between tumor cells and the central nervous system microenvironment, involving blood-brain barrier disruption, immunosuppressive niche formation, neural co-optation, metabolic adaptation, and peripheral immune dysregulation. Current strategies face intrinsic resistance and delivery barriers. This review aimed to systematically examine these mechanisms and therapeutic frontiers, including emerging interventions that preserve vascular-neural integrity, intercept neurotransmitter-mediated tumor support, reprogram brain resident cells, exploit metabolic vulnerabilities, and engineer advanced delivery systems, thereby proposing directions to overcome therapeutic challenges in lung cancer BM.

## 1 Introduction

Lung cancer is a major global health burden and is the leading cause of cancer-related incidence and mortality in China (1–3). It is also the most common source of brain metastases (BM), which are present in 20%–30% of patients with non-small cell lung cancer (NSCLC) at diagnosis and account for 30%–35% of all secondary brain tumors (4, 5). The risk of BM is further increased in patients harboring driver mutations, such as epidermal growth factor receptor (EGFR) or anaplastic lymphoma kinase (ALK) alterations (6). The prognosis of lung cancer patients with BM is generally poor (7). In NSCLC, median survival historically ranges from months to multiple years depending on treatment and molecular subtype, whereas survival is more limited in small cell lung cancer (SCLC), with a median overall survival (mOS) of 8–13 months in extensive-stage disease (8, 9). This is particularly evident for metastases located in critical regions such as the leptomeninges or brainstem.

The development of BM is a complex, multistep process in which the fate of disseminated tumor cells is governed by intricate interactions within the central nervous system (CNS) microenvironment (10, 11). Management of lung cancer BM has now entered an era of multimodal therapy. However, treatment efficacy remains limited by the blood–brain barrier (BBB), intrinsic or acquired therapeutic resistance, and a profoundly immunosuppressive microenvironment. This review aimed to systematically elucidate the interplay between lung cancer cells and both neural and immune components of the CNS microenvironment. It further examines the limitations of conventional therapies and explores emerging strategies targeting neural–immune circuitry.

## 2 The neural-immune landscape in lung cancer BM

**Figure 1:**
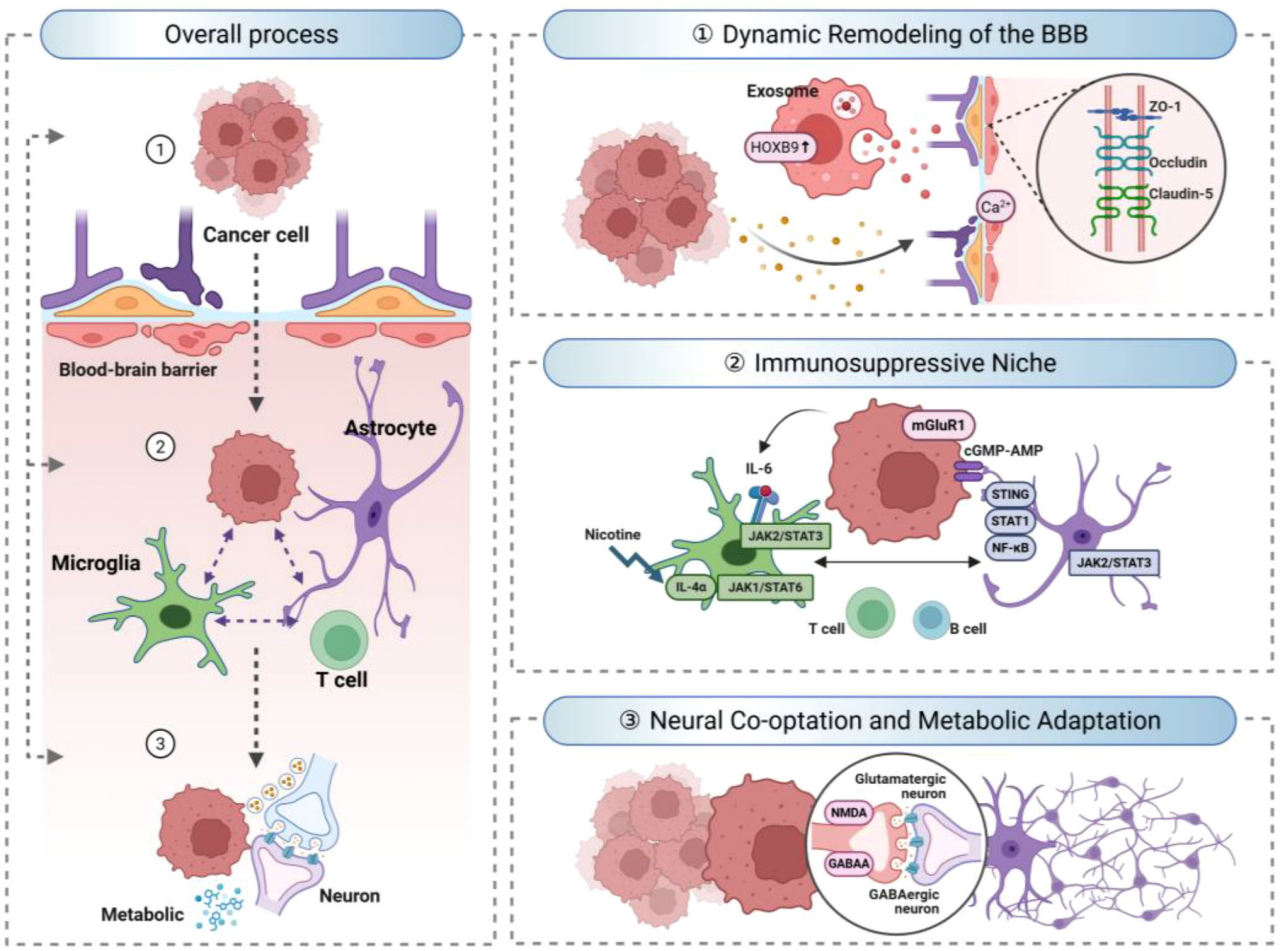
The neural-immune landscape in lung cancer BM. Lung cancer brain metastasis progression involves three interrelated phases: dynamic disruption of the BBB to allow tumor entry, establishment of an immunosuppressive niche through reprogramming of resident glial cells, and hijacking of host neural circuits whereby tumor cells exploit neurotransmitter and neurotrophic signaling for survival and proliferation.

Lung cancer BM progression represents an active, multifaceted remodeling of the CNS microenvironment, rather than merely a passive colonization event. This complex remodeling process encompasses three interrelated aspects: dynamic disruption of the BBB, which facilitates tumor cell entry into the CNS; establishment of an intensely immunosuppressive niche via reprogramming of resident glial cells (11, 12); and commandeering host neural circuits by tumor cells, where they strategically exploit neurotransmitter and neurotrophic signaling to ensure their survival and proliferation. These interconnected processes highlight the critical bidirectional interactions that drive the progression of BM. (**Figure 1**).

### 2.1 Dynamic remodeling of the BBB as a gateway for tumor entry

The BBB is both a physical shield and a vulnerable interface that tumor cells actively remodel to facilitate invasion. Structurally composed of brain microvascular endothelial cells interconnected by tight junction proteins (e.g., ZO-1, Occludin, Claudin-5), supported by pericytes and astrocytic end-feet (13), the BBB maintains CNS homeostasis. In lung cancer BM, circulating tumor cells (CTCs) disrupt this neurovascular unit through coordinated, tumor-derived signals (11).

Tumor-derived exosomes downregulate tight junction proteins by promoting endothelial-mesenchymal transition (EMT) via Transforming Growth Factor-beta (TGF-β) signaling (14) or through exosomal long non-coding RNAs such as LINC01356 (15). Elevated HOXB9 expression in tumor cells induces EMT and matrix metalloproteinase-9 (MMP-9) secretion, degrading inter-endothelial adhesions (16). Clinical evidence (17) further implicates microRNAs (e.g., miR-522-3p) in modulating BBB permeability by directly targeting junction-associated proteins.

Astrocytes play a particularly multifaceted role in modulating BBB permeability. They activate tumor-derived MMPs to remodel the extracellular matrix (18); regulate endothelial integrity through TGF-β, bone morphogenetic protein, and Wnt/β-catenin signaling, and secrete permeability factors including vascular endothelial growth factor (VEGF) and C-X-C motif chemokine ligand 10 (CXCL10), collectively compromising barrier function.

This pro-metastatic cascade is amplified by a feed-forward loop between tumor cells and the neurovascular unit. Lung adenocarcinoma cells secrete acetylcholine and express acetylcholinesterase (AChE) (13). AChE activation induces astrocytic Ca^2+^ overload and adenosine triphosphate (ATP) release, leading to dysfunction and apoptosis that impair BBB integrity.

Thus, rather than a static barrier, the BBB is a dynamically remodeled interface that tumor cells exploit for entry. Subsequent tumor survival and growth depend on reprogramming resident glial and immune cells to transform the parenchyma from a hostile environment into a supportive niche.

### 2.2 Establishment of the immunosuppressive niche

Upon breaching the BBB, tumor cells rely on reprogramming of resident neural cells for survival and proliferation in the brain parenchyma (19)., including microglia, astrocytes, and infiltrating immune cells, to effectively suppress anti-tumor immune responses (20) and establish a supportive microenvironment. This transformation represents an orchestrated, interactive network rather than isolated events.

#### 2.2.1 Microglia and astrocytes are actively re-educated into tumor accomplices

Microglia, the brain’s resident innate immune cells, undergo substantial functional reprogramming in the lung cancer BM microenvironment, shifting from a homeostatic surveillance state to an immunosuppressive and tumor-promoting state that facilitates metastatic colonization (21). This reprogramming is orchestrated by a complex network of tumor-derived signals: soluble factors such as interleukin-6 (IL-6) can directly activate the janus kinase 2 (JAK2)/signal transducer and activator of transcription 3 (STAT3) pathway (22); tumor-derived exosomes containing LINC00482 enhance TGF-β1 expression, reinforcing immunosuppression (23); and external stimuli such as nicotine exposure can further skew microglial function toward a pro-tumor phenotype (24). In addition, cholesterol within the brain microenvironment directly interacts with interleukin-4 receptor α (IL-4Rα) on microglia, promoting its recruitment to lipid rafts and activating janus kinase 1 (JAK1)/signal transducer and activator of transcription 6(STAT6) signaling, which drives upregulation of CD206 and CD163, thereby establishing a permissive pre-metastatic niche (25).

The traditional M1/M2 binary framework has been superseded by a more nuanced understanding of microglial transcriptional states revealed by single-cell sequencing (26). In neurodegenerative diseases, a conserved disease-associated microglia signature has been identified. Whether an analogous metastasis-associated microglia state exists in the BM microenvironment remains to be elucidated; if such a state can be defined, its transcriptional signature could provide new targets for precision therapy. Once reprogrammed, these microglia actively construct an immunosuppressive niche: they secrete interleukin-10 (IL-10), impair cytotoxic T cell function, exhibit reduced phagocytic capacity (27–29), and interact with reactive astrocytes through the macrophage migration inhibitory factor-CD74 complex (MIF-CD74) signaling axis to maintain immunosuppression and promote metastatic progression (30).

In addition to the rapid response mounted by microglia, astrocytes are key architects of the BM microenvironment, participating in the formation of the immunosuppressive niche and mediating multicellular interaction networks through three core mechanisms: direct communication, immunosuppression, and metabolic reprogramming (31). At the level of direct communication: CX43 gap junctions transfer cyclic GMP-AMP (cGAMP) from tumor cells to astrocytes, activating the STING pathway in astrocytes, which in turn produces inflammatory cytokines that act back on tumor cells to promote their growth (32); in bidirectional soluble factor loops, tumor-secreted interleukin-8 (IL-8), MIF, and plasminogen activator inhibitor-1 (PAI-1) induce astrocytes to release IL-6, tumor necrosis factor-alpha (TNF-α), and interleukin-1β (IL-1β), synergistically driving tumor proliferation (33). In the LCN2-SLC22A17 positive feedback loop, tumor-derived LCN2 activates astrocytic JAK2/STAT3 signaling, recruiting macrophages that secrete IL-1β, which further upregulates LCN2 and accelerates metastasis (34). Regarding immunosuppression, astrocytes cooperatively induce T cell apoptosis through pSTAT3-TIMP1, Cdk5-mediated downregulation of major histocompatibility complex class I, and IL-11-driven upregulation of programmed death-ligand 1 (PD-L1), establishing an immune-privileged zone around metastatic lesions (35). Regarding metabolic reprogramming, astrocytes disrupt the glutamate-glutamine cycle and induce tumor cell expression of mGluR1 via the Wnt-5a/PRICKLE1/REST axis, conferring the ability to adapt to the CNS microenvironment through EGFR-dependent glutamate signaling (36, 37). Additionally, astrocytes remodel the extracellular matrix by secreting hyaluronic acid and serpins, and participate in the establishment of the pre-metastatic niche induced by tumor-derived exosomes (38–40). Thus, astrocytes function as a central hub that integrates and amplifies immunosuppressive signals within the metastatic niche.

#### 2.2.2 T cell dysfunction and B cell-mediated immunity

Beyond resident glial cell reprogramming, the adaptive immune compartment undergoes profound functional alterations that collectively reinforce the BM landscape. T cell dysfunction represents a core feature of the immunosuppressive microenvironment in BM. Compared with primary lung tumors, BM lesions exhibit significantly reduced infiltration densities of CD3^+^ and CD8^+^ T cells, alongside selective enrichment of highly suppressive regulatory T cells (Tregs) that overexpress inhibitory receptors including CD25, cytotoxic T lymphocyte-associated antigen-4 (CTLA-4), and programmed cell death protein 1(PD-1), collectively impairing local antitumor immunity (41–44). HSP70-high T cells exhibit profound exhaustion and dysfunction, and their higher abundance predicts shorter survival after PD-1 blockade (45). Tumor cells actively shape this immunosuppressive landscape by modulating downstream effectors such as IL-11 and chemokine (C-C motif) ligand 2 (CCL2) via nuclear factor of activated T cells (NFAT) signaling (46). Spatial transcriptomic analyses further reveal that cytotoxic T cells are physically confined to stromal regions and excluded from tumor cores, establishing a spatial immune exclusion architecture reinforced by immunosuppressive cells such as microglia (47). Despite this suppressive microenvironment, the immune landscape of BM exhibits marked heterogeneity. Notably, the formation of tertiary lymphoid structures (TLS) containing CXCL13^+^ CD4^+^ T cells and B cell aggregates correlates strongly with favorable prognosis, indicating that protective antitumor immunity can be locally activated even within the highly immunosuppressive BM microenvironment (48). Single-cell sequencing further reveals clonally expanded B cells, underscoring their underappreciated yet critical role in lung adenocarcinoma BM (49).

Nonetheless, the overall immunosuppressive microenvironment ultimately facilitates tumor cell survival, proliferation, and metastatic outgrowth in the brain.

### 2.3 Neural co-optation and metabolic adaptation

Complementary to sculpting an immunosuppressive niche, metastatic lung cancer cells further secure their foothold through two interconnected drivers: neural co-optation, which involves active integration into host neural circuits; and metabolic reprogramming that enables adaptation to the CNS microenvironment. At the level of neural co-optation, metastatic lung cancer cells integrate into host neural circuits through multi-scale mechanisms. Upon entering the brain parenchyma, tumor cells undergo transcriptional reprogramming, acquiring transcriptional states resembling neural progenitor cells (NPCs) and oligodendrocyte progenitor cells (OPCs) (49), while upregulating neurotransmitter receptor genes (e.g., *GRIA2*, *GRIN2B*, *GRM4*) and synaptic mediators (e.g., NRXN1, NGFR), thereby gaining the capacity to receive neuronal inputs (50). Subsequently, tumor cells establish functional synaptic connections with neurons, particularly pronounced in SCLC, wherein glutamatergic and GABAergic neurons deliver pro-proliferative signals via N-methyl-D-aspartate (NMDA) and gamma-aminobutyric acid type A (GABAA) receptors; notably, GABAA receptor activation in this context elicits depolarizing currents, promoting tumor cell survival (51, 52). Furthermore, neural signals remodel the metastatic niche by modulating vascular function: nerve growth factor (NGF) binding to endothelial TrkA receptors exerts biphasic effects on tight junction protein expression, preserving BBB integrity during early metastasis to shelter dormant tumor cells, while disrupting barrier function at later stages to facilitate dissemination (53).

With regard to metabolic adaptation, BM cells exhibit profound reprogramming of glucose, amino acid and lipid metabolism. SEC61G inhibits PGAM1 degradation, thereby enhancing glycolysis and oxidative phosphorylation (27). Reprogramming of amino acid metabolism, including serine and glycine, promotes tumor cell migration into the brain through SHMT1-dependent pathways (54, 55). Mitochondrial metabolic programs are preferentially activated and frequently coincide with immunosuppressive microenvironment remodeling (56). Furthermore, lipid metabolism is increasingly recognized as a key driver of brain colonization. The insulin-like growth factor 2 mRNA-binding protein 3 (IGF2BP3)-fatty acid synthase (FASN) axis drives lipid metabolic reprogramming, promoting non-small cell lung cancer brain colonization (57).

These two processes exhibit functional synergy: neural activity modulates tumor cell metabolic adaptation through glutamate metabolism (37), while metabolic reprogramming equips tumor cells with the capacity to adapt to the unique nutrient constraints of the CNS microenvironment.

Thus, BBB remodeling, immunosuppressive niche formation, and neural co-optation should be viewed not as sequential steps but as an integrated network whose components reinforce one another to establish a durable metastatic ecosystem.

## 3 Peripheral immunity: systemic dysregulation in BM

**Figure 2:**
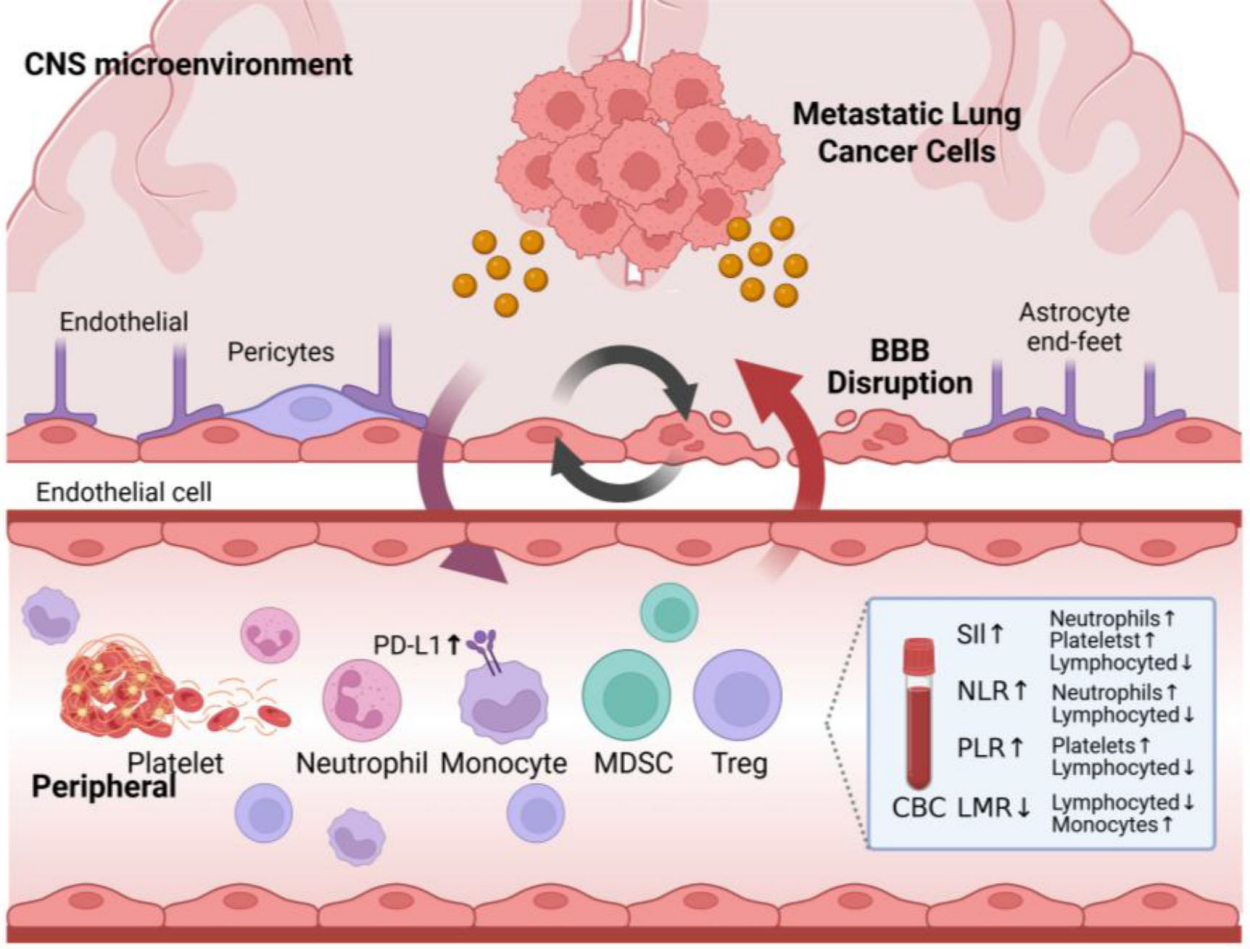
Schematic diagram of the mechanism of peripheral blood immune imbalance in brain metastasis from lung cancer. Patients with lung cancer BM exhibit a pronounced state of systemic immunosuppression in the peripheral blood. This immune imbalance facilitates tumor cell intracranial colonization by promoting the expansion of immunosuppressive cells and the release of inflammatory mediators, thereby increasing BBB permeability.

Beyond the local reprogramming of the CNS microenvironment, lung cancer BM is associated with profound systemic immunosuppression, which actively contributes to tumor cell invasion, BBB disruption, and intracranial colonization. From a neural-immune perspective, these systemic alterations can be viewed as an extension of the tumor’s remodeling influence. (**Figure 2**).

### 3.1 Inflammatory indices as prognostic signals of systemic dysregulation

Comprehensive blood count (CBC)-derived inflammatory indices offer a readily accessible window into this systemic dysregulation. The systemic immune-inflammatory index (SII), neutrophil-to-lymphocyte ratio (NLR), platelet-to-lymphocyte ratio (PLR), and lymphocyte-to-monocyte ratio (LMR) have demonstrated significant clinical utility in BM. Clinical studies have validated these indices specifically in the BM context. Elevated NLR and reduced LMR are associated with an increased risk of BM in NSCLC patients, while high NLR, PLR, or SII correlate with worse prognosis following a BM diagnosis (58–60). These indices provide a practical, cost-effective tool for risk stratification, reflecting the systemic inflammatory state that accompanies and facilitates the neural-immune remodeling central to metastatic progression.

### 3.2 Phenotypic and functional immune alterations in the periphery

Beyond quantitative shifts, patients with lung cancer BM exhibit distinct phenotypic alterations in peripheral immune populations that mirror the immunosuppressive landscape of the CNS (61, 62). These include increased PD-L1 expression on monocytes, expansion of myeloid-derived suppressor cell (MDSC) and Treg populations, and a dysfunctional T cell profile marked by impaired effector memory differentiation (62–65). Tumor-derived factors, such as IL-6, are implicated in inducing these PD-L1^+^ myeloid cells, suggesting a direct mechanistic link between tumor burden and systemic immune suppression (63).

These systemic immune alterations are closely linked to the CNS microenvironment. This peripheral-immune connection offers new therapeutic opportunities, suggesting that strategies aimed at normalizing systemic immunity may enhance the efficacy of CNS-directed immunotherapies. Future research should focus on identifying peripheral biomarkers that predict response to such therapies and on determining whether dynamic changes in these markers can inform treatment duration and combination strategies.

## 4 Traditional therapies for lung cancer BM

**Figure 3:**
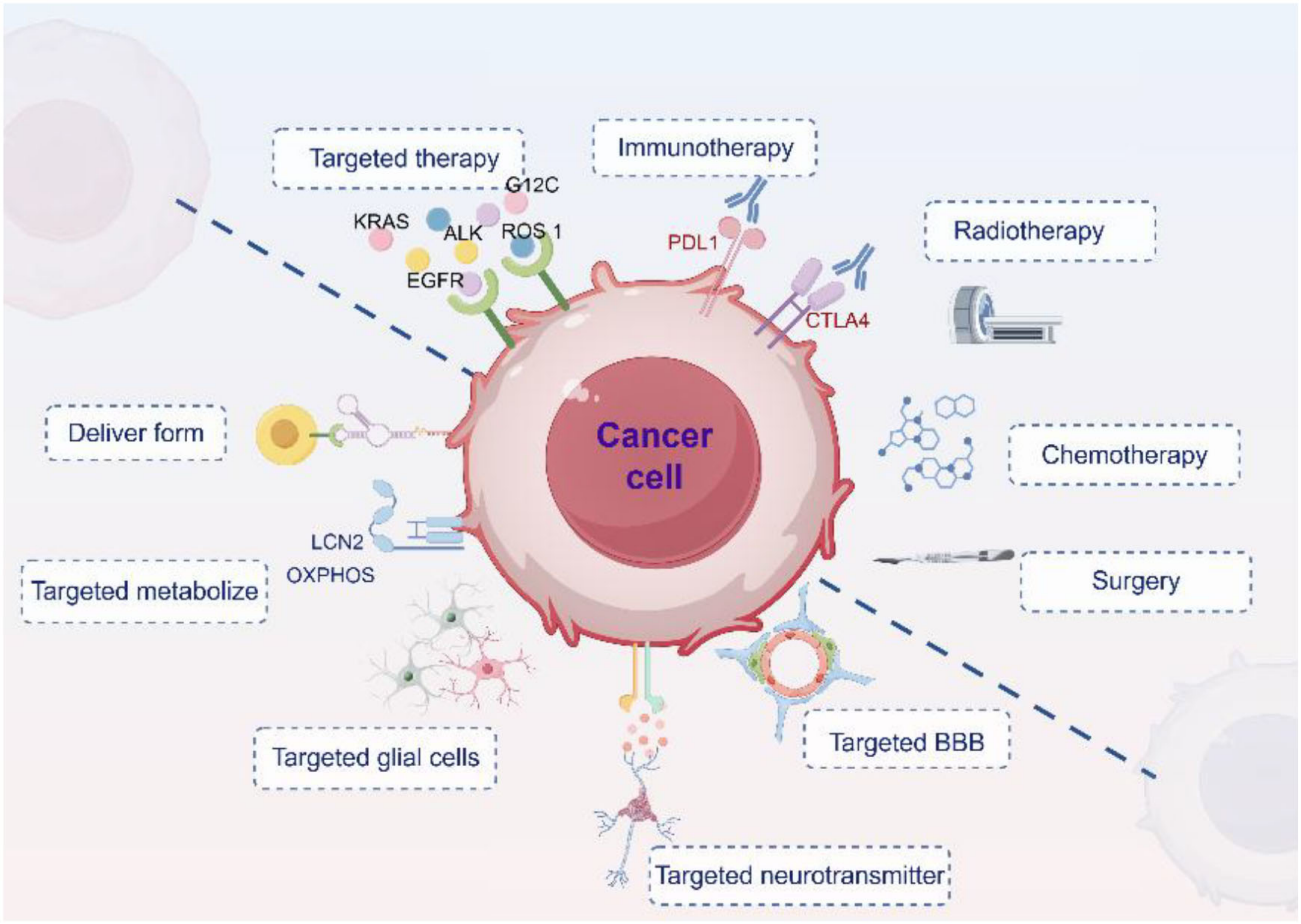
Therapies for lung cancer brain metastasis. Conventional modalities include targeted therapy, immunotherapy, radiotherapy, chemotherapy, and surgery. Emerging strategies focus on the neural–immune microenvironment, including optimization of drug delivery forms, targeting tumor metabolism, glial cells, neurotransmitter signaling, and the BBB.

BM management has progressed from single treatments to combined approaches, improving local control and survival. Surgery is an option for limited metastases but carries neurological risks. Radiotherapy (WBRT, SRS) remains standard, with prophylactic cranial irradiation recommended for responsive SCLC (66) though neurocognitive decline limits its palliative benefit. Chemotherapy is crucial for SCLC and driver-negative NSCLC, but the BBB restricts drug delivery, yielding intracranial response rates of only 15-50% (67) and mOS of just 3–6 months (68).(**Figure 3**).

Targeted therapy revolutionized driver-positive NSCLC treatment by overcoming this barrier through rational drug design. EGFR/ALK inhibitors demonstrate significant intracranial efficacy (69–71). New-generation Tyrosine Kinase Inhibitors (TKIs) like Zorifertinib significantly improves intracranial Progression-Free Survival (PFS) vs. first-gen TKIs and shows potential for extended OS in sequential therapy (72). The ACHIEVE trial showed high-dose aumolertinib achieved an objective response rate (ORR) of 82.5% in BM (73). CNS progression and acquired resistance persist (74), underscoring that even optimal BBB penetration cannot eliminate adaptive resistance. Anti-angiogenics like bevacizumab operate through a complementary mechanism: inhibiting VEGF not only exerts direct antitumor effects but also alleviates post-radiotherapy edema and may transiently modulate BBB permeability to enhance drug delivery (75).

Immunotherapy has fundamentally revised the view of the CNS as immune-privileged by demonstrating that T cells can be reactivated within the immunosuppressive BM microenvironment (76). Immune checkpoint inhibitors (ICIs) extend mOS in metastatic NSCLC to 18–24 months (1), with KEYNOTE subgroup analyses confirming the superiority of ICIs over chemotherapy in BM patients (77); chemoimmunotherapy improves OS and PFS versus chemotherapy alone (78). Dual ICIs regimens (nivolumab+ipilimumab) are first-line for extensive-stage small cell lung cancer, delaying brain progression (79, 80). The C-Brain study confirmed efficacy and safety of brain radiotherapy+camrelizumab+chemotherapy in untreated NSCLC BM (81). However, immunotherapy resistance and immune-related adverse events remain significant limitations, underscoring the need to better integrate therapeutic development with our understanding of the mechanisms that govern T cell dysfunction within the metastatic niche.

## 5 Novel strategies targeting the neural-immune circuitry

### 5.1 Preserving vascular-neural interface integrity to prevent metastatic entry

The BBB is not merely a passive obstacle but a dynamic interface that tumor cells actively remodel to facilitate invasion. In the context of preventing or limiting early dissemination, strategies aimed at restoring and preserving BBB integrity may block tumor cell entry or suppress the formation of a permissive pre-metastatic niche. Tumor-derived complement component 3 (C3) activates C3a receptor signaling in the choroid plexus, compromising blood-cerebrospinal fluid barrier integrity and enabling leptomeningeal dissemination; pharmacological C3a receptor blockade prevents this process in preclinical models (82). Similarly, CD276 is consistently overexpressed on BM-associated endothelial and mural cells; antibody-mediated CD276 blockade suppresses tumor growth and enhances CD8^+^ T cell infiltration into metastatic lesions (83). Together, these approaches reframe the barrier as a therapeutic target, restoring its integrity to deny tumor cells access and deprive established metastases of vascular support. (**Table 1**).

**Table 1:** Novel strategies targeting the neural-immune circuitry.

Strategy	Target	Agent	Clinical stage	Key mechanism/Results	Ref
Preserving vascular-neural interface integrity	C3aR	C3aR antagonist	Preclinical	Blocks tumor-derived C3-induced blood-CSF barrier disruption, preventing leptomeningeal metastasis	(82)
	CD276	Anti-CD276 antibody	Preclinical	Enhances CD8^+^ T cell infiltration into BM lesions, reduces tumor growth	(83)
Intercepting neurotransmitter support	STAT6/GABA	STAT6 inhibitor (AS1517499)	Preclinical	Blocks IL-13-driven STAT6 activation in choroid plexus, reducing CSF GABA and metabolic support for tumor cells	(84)
	Glutamate	Riluzole	Preclinical	Inhibits glutamate release, disrupts paracrine signaling, delays tumor progression, enhances chemotherapy	(52)
	Glutamate	Levetiracetam	Preclinical	Disrupts synaptic vesicle release, reduces tumor burden	(51)
Reprogramming Brain-Resident Cells	HSP47	Col003	Preclinical	Reverses immunosuppressive microglial phenotype, restores CD8^+^ T cell immunity, enhances anti-PD-L1 efficacy	(29)
	IFITM1	IFITM1-enriched oncolytic virus	Preclinical	Activates microglia and CD8^+^ T cells via MHC-I upregulation; synergizes with PD-1 blockade to prevent BM	(85)
	IL-11 gp130/EGFR	gp130/EGFR dual inhibitor	Preclinical	Blocks astrocyte-derived IL-11 signaling, reverses PD-L1-mediated CD8^+^ T cell apoptosis	(35)
	STAT3	Silibinin	Phase II	Suppresses STAT3/NF-κB in tumor cells and astrocytes; reduces tumor-astrocyte crosstalk and vascular permeability	(86)
	GAS6-AXL-CD146	Imaprelimab (anti-CD146), Bemcentinib (AXL inhibitor)	Preclinical	Disrupts reactive astrocyte-driven GAS6-AXL signaling, inhibits cancer stem cell pericyte-like mimicry and VEGF signaling	(87)
Exploiting metabolic vulnerabilities	OXPHOS	Gamitrinib	Preclinical	Induces apoptosis in BM lesions; synergizes with anti-PD-1 to enhance immune infiltration	(56)
	LCN2/SLC22A17	Iron chelation therapy	Preclinical	Blocks tumor cell iron uptake, inhibits leptomeningeal metastasis	(88)
	FASN	TVB-2640	Preclinical	Inhibits palmitate synthesis, reduces LOX expression and cytoskeleton remodeling	(89)
Engineering the delivery interface	HER2	T-DXd	Phase II	In the 5.4 mg/kg cohort, the cORR in patients with CNS metastases at baseline was 60.0%	(90)
	HER3	HER3-DXd	Phase II	33.3% intracranial ORR in patients with NSCLC BM who had not received prior brain radiotherapy	(91)
	TROP2	Dato-DXd	Phase III	38% CNS cORR in NSCLC BM	(92)
	BBB tight junctions/transcytosis	Afa/LPN-FD7 nanoparticles	Preclinical	Disrupts tight junctions and activates transcytosis to enhance BBB crossing	(94)
	LPCAT1	ExoscFv/siLPCAT1 (EGFR-targeting exosomes)	Preclinical	Delivers siRNA to silence LPCAT1, inhibits PI3K/AKT/MYC pathway	(95)
	Astrocyte-tumor gap junctions	LAsomes (CBX-loaded biomimetic liposomes)	Preclinical	Blocks connexin-mediated Ca²^+^ and cGAMP transfer, reverses chemoresistance	(96)
	USP7	P5091@RMPs-R4F (SR-B1-targeting microparticles)	Preclinical	Reprograms M2 macrophages and enhances T cell infiltration	(97)

C3aR, complement component 3a receptor; CD276, cluster of differentiation 276; STAT6, signal transducer and activator of transcription 6; GABA, gamma-aminobutyric acid; HSP47, heat shock protein 47; IFITM1, interferon induced transmembrane protein 1; IL-11, interleukin 11; gp130, glycoprotein 130; EGFR, epidermal growth factor receptor; STAT3, signal transducer and activator of transcription 3; NF-κB, nuclear factor kappa-light-chain-enhancer of activated B cells; GAS6, growth arrest specific 6; CD146, cluster of differentiation 146; OXPHOS, oxidative phosphorylation; LCN2, lipocalin 2; SLC22A17, solute carrier family 22 member 17; FASN, fatty acid synthase; HER2, human epidermal growth factor receptor 2; T-DXd, trastuzumab deruxtecan; cORR, confirmed objective response rate; BM, brain metastasis; HER3, human epidermal growth factor receptor 3; HER3-DXd, HER3-directed deruxtecan; TROP2, trophoblast cell surface antigen 2; Dato-DXd, datopotamab deruxtecan; BBB, blood-brain barrier; LPCAT1, lysophosphatidylcholine acyltransferase 1; PI3K, phosphoinositide 3-kinase; AKT, protein kinase B; MYC, MYC proto-oncogene; USP7, ubiquitin specific peptidase 7; VEGF, vascular endothelial growth factor; PD-L1, programmed death-ligand 1; MHC-I, major histocompatibility complex class I; cGAMP, cyclic guanosine monophosphate-adenosine monophosphate; SR-B1, scavenger receptor class B type 1; CSF, cerebrospinal fluid.

### 5.2 Intercepting neurotransmitter-mediated tumor support

Tumor cells utilize neurotransmitter signaling through two mechanisms: metabolic dependency and direct synaptic communication. In the nutrient-restricted leptomeningeal space, metastatic cells depend on GABA as a metabolic substrate. Tumor-derived interleukin-13 (IL-13) activates STAT6 signaling in the choroid plexus, increasing cerebrospinal fluid GABA levels and thereby supporting tumor growth (84). Disruption of this pathway, via IL-13 signaling blockade or GABA metabolism inhibition, may eliminate this metabolic support.

Direct neuron-tumor synaptic communication represents a therapeutic target, particularly in SCLC. Glutamatergic signaling drives SCLC-neuron interactions. The GRM8 agonist DCPG and the glutamate release inhibitor riluzole delayed tumor progression and improved survival, with riluzole also enhancing chemotherapy efficacy (52). Separately, the anti-epileptic drug levetiracetam, which disrupts synaptic vesicle release, reduced tumor burden and inhibited cancer cell growth in SCLC BM models (51). These findings support repurposing of FDA-approved neuromodulatory drugs for brain metastasis, given their established safety profiles and CNS penetration. (**Table 1**).

### 5.3 Reprogramming brain-resident cells

Microglia and astrocytes, once reprogrammed by tumor-derived signals, become active accomplices in metastatic progression. Emerging strategies seek to re-educate these cells toward tumor-suppressive states rather than depleting them. The small molecule inhibitor Col003 disrupts HSP47-mediated collagen deposition, reversing the immunosuppressive microglial phenotype and restoring CD8^+^ T cell immunity (29). Conversely, IFITM1 activates microglia and boosts CD8^+^ T cell responses; an IFITM1-enriched oncolytic virus synergizes with PD-1 blockade to prevent BM (85).

Astrocyte-targeted strategies have evolved similarly. Dual targeting of IL-11 and EGFR signaling reduces metastatic growth and reverses immune escape (35). The STAT3 inhibitor silibinin, currently in Phase II trials for preventing BM recurrence (NCT05689619), targets this pathway in both tumor cells and astrocytes (86). Notably, reactive astrocytes upregulate CD146 expression via the GAS6-AXL axis, and targeting this pathway with the anti-CD146 antibody Imaprelimab or the AXL inhibitor Bemcentinib each outperformed bevacizumab in preclinical models, suggesting that disrupting tumor-stroma crosstalk may be more effective than VEGF blockade alone (87). (**Table 1**).

### 5.4 Exploiting metabolic vulnerabilities in BM

Metabolic reprogramming enables tumor cells to survive within the nutrient-restricted brain microenvironment. Oxidative phosphorylation (OXPHOS) is enriched in BM lesions and coincides with immune suppression; the OXPHOS inhibitor gamitrinib synergizes with anti-PD-1 immunotherapy (56). Through the LCN2-SLC22A17 axis, tumor cells outcompete macrophages for iron in the cerebrospinal fluid; iron chelation therapy disrupts this competition and inhibits leptomeningeal metastasis (88). Lipid metabolism represents an additional vulnerability: The FASN inhibitor TVB-2640 targets palmitate synthesis, reducing LOX expression and inhibiting cytoskeleton remodeling essential for metastatic dissemination (89). (**Table 1**).

### 5.5 Engineering the delivery interface

Effective treatment of established BM requires therapeutic agents to cross the BBB. Emerging delivery strategies exploit the biological features of the metastatic niche rather than attempting passive penetration.

Antibody-drug conjugates (ADCs) combine tumor-targeting antibodies with cytotoxic payloads. Several ADCs have demonstrated intracranial activity in lung cancer BM. The HER2-targeting ADC T-DXd achieved a 50% intracranial ORR in the DESTINY-Lung02 trial (90). The HER3-targeting ADC HER3-DXd showed a 33.3% intracranial ORR in HERTHENA-Lung01 (91), while the TROP2-targeting ADC Dato-DXd reported a 38% ORR in BM (92). However, as macromolecules (~150 kDa), ADCs exhibit limited penetration of an intact BBB, restricting their efficacy to lesions with pre-existing barrier disruption. Additional challenges include suboptimal pharmacokinetics, off-tumor toxicity, and acquired resistance (93).

To overcome these limitations, next-generation delivery platforms are being designed to actively engage with the cellular and molecular landscape of the metastatic niche. These strategies include nanoparticles that disrupt tight junctions while activating transcytosis to enhance BBB crossing (94); EGFR-targeting exosomes that deliver siRNA against LPCAT1, thereby inhibiting the PI3K/AKT/MYC pathway in tumor cells (95); biomimetic liposomes (LAsomes) derived from fusing membranes of reactive astrocytes and brain metastatic cells, which target both tumor cells and adjacent astrocytes to disrupt gap junction-mediated communication (96); and SR-B1-targeting microparticles that deliver a USP7 inhibitor to reprogram M2 macrophages and enhance T cell infiltration (97).

These approaches, while largely preclinical, illustrate a conceptual shift from passive BBB penetration to active engagement with niche-specific targets. ADCs represent an initial clinical application of this principle, and their integration with advanced engineering strategies may improve outcomes for patients with lung cancer BM. (**Table 1**).

## 6 Future perspectives

**Figure 4:**
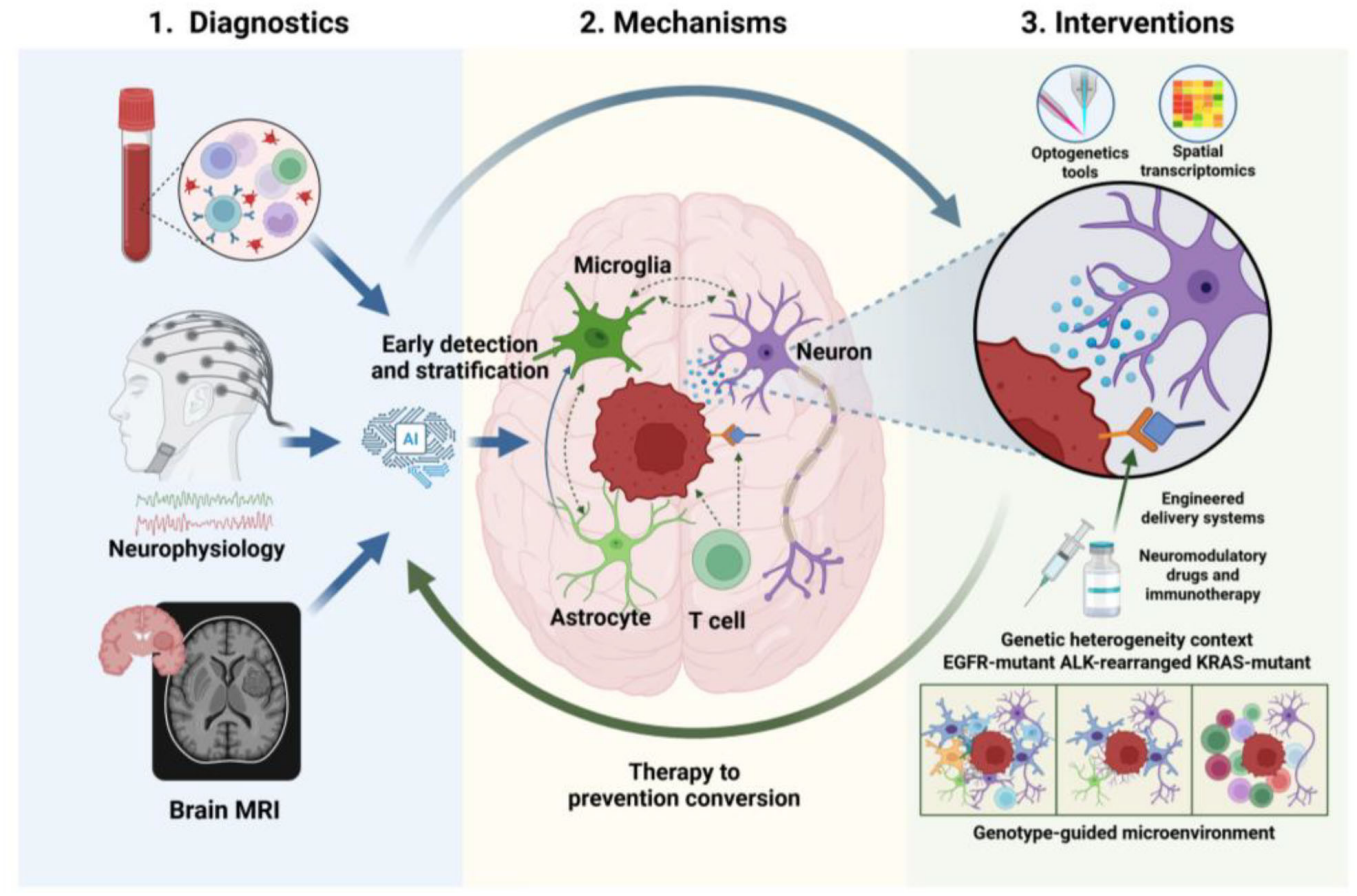
Future diagnosis and treatment strategies for lung cancer brain metastasis Future directions in lung cancer BM research call for an integrated neural-immune framework that moves beyond reactive treatment toward proactive interception, leveraging multi-dimensional biomarkers and AI integration for pre-symptomatic diagnosis. Translating these insights requires elucidating the neural-immune axis mechanistically, developing humanized preclinical models that recapitulate central nervous system heterogeneity across genetic subtypes, and designing genotype-guided therapeutic strategies that target neural-immune crosstalk, ultimately aiming to prevent metastasis by intercepting the neural-immune circuits that sustain the metastatic niche.

Lung cancer BM represents a multifaceted challenge that extends beyond tumor cell biology to encompass the dynamic interplay between neural and immune circuits within the CNS. The emerging field of cancer neuroscience provides a conceptual framework for reimagining these challenges as opportunities for intervention, shifting the paradigm from reactive treatment toward proactive interception of metastatic progression. (**Figure 4**).

### 6.1 New diagnostic approaches

Current diagnostic approaches rely heavily on neuroimaging, detecting metastases only after they reach clinically apparent size and thereby missing a critical window for early intervention (98). The recognition that molecular, neural, and immune alterations precede overt metastasis opens new diagnostic possibilities through three complementary dimensions of peripheral biomarkers: direct tumor-derived markers (CTCs, ctDNA, DTCs) reflecting real-time tumor burden and clonal evolution; neural markers indicating tumor-induced neuronal injury; and immune cell subsets and cytokine profiles (99), along with immune-inflammatory indices capturing systemic dysregulation (100).

Emerging evidence further suggests that neurophysiological techniques such as electroencephalography can detect functional disturbances associated with leptomeningeal dissemination, with 10%-30% of patients experiencing seizures prior to radiographic diagnosis (101). Integration of patient-reported outcomes through validated instruments such as the European Organization for Research and Treatment of Cancer Quality of Life Questionnaire-Brain Cancer Module 20 (EORTC QLQ-BN20) (102) and Generalized Anxiety Disorder-7 (GAD-7) (103) scales adds a critical patient-centered dimension. The integration of these multimodal data streams, imaging, electrophysiology, circulating biomarkers, and clinical parameters, through artificial intelligence and machine learning frameworks holds promise for developing diagnostic models that identify patients at highest risk, potentially enabling earlier intervention. However, realizing this potential will require not only robust predictive algorithms but also the development of safe and effective preventive strategies (104).

### 6.2 Elucidating and targeting neural-immune crosstalk

Several fundamental questions remain unanswered regarding neural-immune crosstalk in lung cancer BM. First, how primary tumors remotely prime the brain’s pre-metastatic niche via humoral routes or direct neural signaling remains unclear. Second, the timing of tumor-neuron synaptic formation (early colonization vs. late adaptation) has critical implications for therapeutic windows. Third, region-specific determinants of metastatic susceptibility, neuronal subtypes, glial densities, neurotransmitter milieus, remain poorly defined but may reveal targetable vulnerabilities. Fourth, neural-immune crosstalk at the synaptic level, specifically how neurotransmitters modulate immune cells and immune signals influence neurons, represents an uncharted frontier requiring advanced models. Emerging technologies including optogenetics, chemogenetics (105), spatial transcriptomics, intravital imaging and microphysiological systems (MPS) (106) are beginning to address these questions. These mechanistic insights are already pointing to therapeutic opportunities, including repurposing neuromodulatory drugs (riluzole, levetiracetam), targeting neural-immune mediators (107), reprogramming brain-resident cells, and engineering niche-exploiting delivery systems.

Beyond these universal questions, an additional layer of complexity arises from genetic heterogeneity (108). EGFR-mutant, ALK-rearranged, and Kirsten rat sarcoma viral oncogene (KRAS)-mutant lung cancers exhibit distinct intracranial tropism and sculpt fundamentally different neural-immune landscapes upon colonizing the brain (108). Elucidating how driver mutations shape the neural-immune architecture of the metastatic niche will be essential for moving toward genotype-guided targeting, wherein therapeutic strategies are tailored not only to tumor cell-intrinsic vulnerabilities but also to the distinctive microenvironmental ecosystems they create (109).

Moreover, targeted therapies, immunotherapy, and radiotherapy each induce distinct remodeling of neural and immune compartments, potentially creating adaptive resistance or novel vulnerabilities (109). Future strategies must account for these shifts by monitoring dynamic changes in neural-immune signatures to guide tailored interventions.

### 6.3 From discovery to clinical translation

Translating these insights into clinical benefit will require parallel advances across multiple interdependent fronts. Biomarker development must move beyond single markers to integrated signatures that capture neural, immune, metabolic, and genetic dimensions of metastatic risk and therapeutic response. Such signatures should be prospectively validated in well-annotated clinical cohorts stratified by driver mutation status and incorporated into routine clinical decision-making. Preclinical models (110) must better recapitulate the complexity of the human CNS and its heterogeneity across genetic contexts, including humanized chimeric systems, patient-derived xenografts representing diverse genetic subtypes, brain organoids with functional vasculature and immune components, and co-culture systems preserving tumor-stromal interactions. Trial design should incorporate neural-immune monitoring and genetic stratification to identify patients most likely to benefit from targeted interventions and to detect early signals of efficacy or toxicity, with adaptive designs enabling biomarker-based stratification and real-time treatment modification.

### 6.4 From treatment to prevention: a new paradigm

We envision a future where the diagnosis of lung cancer triggers not just a search for existing metastases, but a proactive strategy to prevent their formation entirely. This vision, closing the loop from precision diagnosis to precision prevention, requires reconceptualizing BM not as a late-stage event but as a process that can be intercepted at multiple points along its trajectory. Realizing this vision demands integrating our understanding of genetic driver diversity, neural-immune heterogeneity, and microenvironmental dynamics into a unified framework. The emerging discipline of cancer neuroscience provides both the conceptual framework and the experimental toolkit for realizing this vision. For patients with lung cancer at risk for or living with BM, the translation of these insights into clinical practice, tailored to the specific genetic and neural-immune architecture of their disease, represents the true promise of this rapidly advancing field.

## 7 Conclusions

Lung cancer BM is driven by dynamic interactions between tumor cells and the CNS microenvironment, involving BBB disruption, reprogramming of glial and immune cells into an immunosuppressive niche, and hijacking of neural circuits alongside metabolic adaptation. Systemic immune dysregulation further contributes to tumor progression. Current therapeutic strategies have improved intracranial disease control but are constrained by the BBB, resistance, and the immunosuppressive niche. Emerging approaches targeting the neural-immune circuitry, such as stabilizing the vascular-neural interface, intercepting neurotransmitter-mediated tumor support, reprogramming brain-resident cells, exploiting metabolic vulnerabilities, and engineering advanced delivery systems, offer new opportunities to overcome these limitations. Future efforts should prioritize early diagnosis through multimodal biomarkers, elucidate neural-immune crosstalk mechanisms, and develop genotype-guided strategies. Translating these insights into practice aims to shift the paradigm from treating established metastases toward proactive interception, improving outcomes and quality of life for patients with lung cancer BM.
